# Urinary Copper Is Associated with Dyslipidemia, and This Association Is Mediated by Inflammation

**DOI:** 10.1007/s12011-025-04581-6

**Published:** 2025-04-02

**Authors:** Sisi Xie, Zoltan Kutalik, Aurélien Thomas, Maïwenn Perrais, Julien Vaucher, Pedro Marques-Vidal

**Affiliations:** 1 Department of Medicine, Internal Medicine, Lausanne University Hospital (CHUV) and University of Lausanne, Rue du Bugnon 46, 1011 Lausanne, Switzerland; 2 Department of Computational Biology, University of Lausanne, 1015 Lausanne, Switzerland; 3 Center for Primary Care and Public Health, University of Lausanne, 1010 Lausanne, Switzerland; 4 Swiss Institute of Bioinformatics, 1015 Lausanne, Switzerland; 5 Unit of Forensic Chemistry and Toxicology, University Centre of Legal Medicine Lausanne-Geneva, Geneva University Hospital and University of Geneva, Rue Michel-Servet 1, 1211 Geneva, Switzerland; 6 Faculty Unit of Toxicology, University Centre of Legal Medicine Lausanne-Geneva, Lausanne University Hospital and University of Lausanne, Chemin de La Vulliette 4, 1000 Lausanne, Switzerland; 7 Department of Internal Medicine and Specialties, Internal Medicine, Fribourg Hospital and University of Fribourg, Fribourg, Switzerland

**Keywords:** Copper, Dyslipidemia, C-reactive protein, Mediation analysis

## Abstract

Dyslipidemia is an important public health issue. Copper may influence lipid metabolism, possibly via inflammation, but the mechanisms remain unclear. This study aimed to assess the association between urinary copper concentrations and blood lipids (total cholesterol (TC), low-density lipoprotein cholesterol (LDL-C), high-density lipoprotein cholesterol (HDL-C), and triglycerides (TG)), and the possible mediating role of inflammation, assessed via high-sensitivity C-reactive protein (hs-CRP). We conducted a cross-sectional, population-based study using baseline data from Switzerland’s CoLaus|PsyCoLaus cohort. Urinary copper was measured from spot urine using inductively coupled plasma mass spectrometry and adjusted for creatinine. Lipid markers and hs-CRP were measured using standardized biochemical assays. Multiple linear regression assessed associations, and mediation effects were evaluated using the SGmediation2 package. A total of 6284 adults (mean age 52.6 years, 53.4% female) were included. Urinary copper was positively associated with TG (beta=0.08, 95%CI 0.04, 0.12) and negatively associated with HDL-C (− 0.04, 95%CI − 0.07, − 0.003). Additionally, urinary copper was positively associated with hs-CRP (0.51, 95%CI 0.42, 0.60), which in turn was positively associated with TG (0.05, 95%CI 0.04, 0.06) and negatively associated with HDL-C (− 0.04, 95%CI − 0.05, − 0.03). Mediation analysis revealed that urinary copper exerts partial indirect effects on TG (mediation effect 31.4%) and HDL-C (56.9%) through hs-CRP. hs-CRP partially mediated the associations between urinary copper and HDL-C and TG, with a robust effect for TG but statistical uncertainty for HDL-C. No mediation was observed for TC or LDL-C. These findings suggest hs-CRP's role in lipid metabolism, especially in TG regulation.

**Graphical Abstract:**

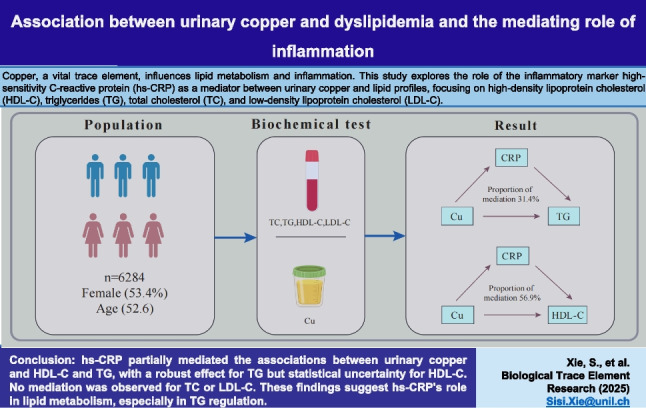

**Supplementary Information:**

The online version contains supplementary material available at 10.1007/s12011-025-04581-6.

## Introduction

Dyslipidemia is a serious global health problem, combining elevated levels of total cholesterol (TC), low-density lipoprotein cholesterol (LDL-C), and triglycerides (TG) and decreased levels of high-density lipoprotein cholesterol (HDL-C) [1]. From 1990 to 2019, the absolute annual burden of death and of disability-adjusted life years attributable to high LDL-C increased by 46% and 41%, respectively [2]. Dyslipidemia is one of the key risk factors for cardiovascular disease, especially atherosclerotic cardiovascular disease, and mortality [3]. The specific cause of dyslipidemia is still unclear. In addition to genetic background, age, and modifiable risk factors (obesity, smoking, unhealthy diet, or physical activity) [4], increasing evidence suggests that environmental trace element exposure may play an important role in abnormal lipid distribution [5, 6].

Copper is an important trace element that participates in various processes of the body, including cell and energy metabolism, oxygen transport, signal transduction, and blood coagulation [7]. Previous studies have found that increased blood or urine copper levels are associated with increased blood lipids [8–10]. However, the choice of biomarker for copper status remains a critical consideration. While blood copper is tightly regulated by homeostatic mechanisms and influenced by acute physiological changes, urinary copper serves as an alternative biomarker that reflects both exposure and excretion pathways. Unlike blood copper, which primarily represents the systemic copper distribution, urinary copper may better capture variations in copper metabolism and elimination, making it a useful marker in large-scale epidemiological studies.

Despite these associations, the biological mechanism linking copper to dyslipidemia remains unclear. Several studies have reported that copper levels are positively correlated with the inflammatory marker C-reactive protein (CRP) [11–13], and other studies have established a link between elevated levels of inflammatory markers and dyslipidemia [14–16]. Hence, a plausible mechanism could be that copper induces dyslipidemia via inflammatory pathways.

Therefore, we conducted a cross-sectional study using data from the CoLaus|PsyCoLaus cohort, a well-characterized, population-based study designed to investigate cardiovascular risk factors. This dataset provides comprehensive biomarker measurements, including lipid profiles, hs-CRP, and urinary copper levels. It serves as an ideal resource for examining the potential associations between urinary copper levels and four lipid markers (TC, LDL-C, HDL-C, and TG). Additionally, it allows for an exploration of the mediating role of inflammation, assessed through high-sensitivity C-reactive protein (hs-CRP), in the relationship between urinary copper and these lipid markers.

## Participants and Methods

### Participants and Study Design

We used data from CoLaus|PsyCoLaus (www.colaus-psycolaus.ch), a population-based study initiated in 2003 with 6733 middle-aged participants from Lausanne, Switzerland, to investigate the epidemiology and genetic determinants of cardiovascular risk factors [17]. A total of 6733 participants aged 35–75 years were recruited between 2003 and 2006, with follow-ups conducted in 2009–2012, 2014–2017, and 2018–2021. The study collected comprehensive health data, including demographics, lifestyle factors, clinical measurements, and biological samples. Urinary copper data are only available at baseline (2003–2006); therefore, only baseline data were used in this study.

### Measurement of Urine Copper

Spot urine samples were collected from participants during clinical visits at baseline. Each sample was processed and stored at − 80 °C until analysis. Urine samples (200 µL) were diluted with 1.8 mL of HNO_3_ 1% solution containing 10 ng/mL Rhodium and 10 ng/mL Indium as internal standard [18, 19]. Samples were analyzed using an inductively coupled plasma mass spectrometer (ICP MS, 7800 Series, Agilent). Urinary copper concentration was adjusted for urinary creatinine. Values below the limit of detection (LOD) were replaced by the LOD value divided by 2. The accuracy and precision of the method were routinely assessed using two certified reference materials named ClinCheck (Recipe Chemicals, München, Germany) as internal quality controls (www.recipe.de/en/index.html). Furthermore, external quality control was ensured through the QMEQAS program from the Public Health Expertise and Reference Centre of Québec (Canada) (www.inspq.qc.ca/ctqenglish/eqas/qmeqas/description) and the LAMP program from the Centers for Disease Control and Prevention (USA), which evaluate analytical performance respectively three and four times per year, to validate the accuracy of trace element measurements. This external validation ensures that our copper analysis adheres to international quality standards.

### Measurement of Blood Lipids and C-Reactive Protein

Biological assays were performed by the CHUV Clinical Laboratory on fresh blood samples within 2 h of blood collection. All measurements were conducted in a Modular P apparatus (Roche Diagnostics, Basel, Switzerland). TC was assessed by cholesterol oxidase phenol 4-aminoantipyrine peroxidase. HDL-C by cholesterol oxidase phenol 4-amino antipyrine peroxidase + polyethylene glycol + cyclodextrin, and TG by glycerol phosphate oxidase. LDL-C was computed using the Friedewald formula. hs-CRP was assessed by immunoassay and latex.

### Relevant Covariates

We selected age (years), sex (male/female), education (low/medium/high), marital status (living alone/living in a couple), weekly alcohol consumption (units), smoking (never/former/current), hypertension (yes/no), diabetes (yes/no), body mass index (BMI) (normal/overweight/obese), and physical activity (never, once a week, twice a week).

Education was categorized into high (university), middle (high school), and low (apprenticeship + mandatory). Marital status was defined as living alone (single, divorced, and widowed) or living with a partner. Usual weekly alcohol consumption was self-reported and reported as a number of units (glasses of wine, bottles or cans of beer, and shots of spirits) per week. Smoking was self-reported and categorized as never, former (irrespective of the time since quitting smoking), and current.

Body weight and height were measured with participants barefoot and in light indoor clothes. Body weight was measured in kilograms to the nearest 100 g using a Seca® scale (Hamburg, Germany). Height was measured to the nearest 5 mm using a Seca® (Hamburg, Germany) height gauge. BMI was calculated and categorized as normal (< 25 kg/m^2^), overweight (≥ 25 and < 30 kg/m^2^), and obese (≥ 30 kg/m^2^).

Blood pressure (BP) was measured thrice using an Omron® HEM-907 automated oscillometric sphygmomanometer after at least a 10-min rest in a seated position, and the average of the last two measurements was used. Hypertension was defined by an SBP ≥ 140 mm Hg or a DBP ≥ 90 mm Hg and/or the presence of antihypertensive drug treatment.

Glucose was assessed by glucose dehydrogenase. Diabetes mellitus (DM) was defined as fasting plasma glucose ≥ 7.0 mmol/L and/or the presence of oral hypoglycaemic or insulin treatment.

### Inclusion and Exclusion Criteria

For this study, all participants at baseline were considered eligible. We then excluded participants with missing data on 1) copper, 2) hs-CRP,3) blood lipids, and 4) covariates.

### Statistical Analysis

Statistical analyses were conducted using Stata v.18 (Stata Corp, College Station, TX, USA). Based on the uneven dispersion of urinary copper and plasma hs-CRP, we performed log transformation to normalize continuous variables and then classified these variables into four quartiles (Q1, Q2, Q3, and Q4) as categorical variables. Similarly, TG were log-transformed due to their skewed distribution. Descriptive results were expressed as number of participants (percentage) for categorical variables and as average ± standard deviation or median [interquartile range] for continuous variables. Between-group comparisons were conducted using chi-square for categorical variables and Student’s *t*-test, analysis of variance (ANOVA) or Kruskal–Wallis test for continuous variables.

Multivariable linear regression was used to evaluate the association between 1) urinary copper levels and blood lipid levels, 2) urinary copper levels and hs-CRP, and 3) hs-CRP and blood lipid levels. Mediation analysis was performed using the SGmediation2 package in Stata. The analysis estimated the total effect, direct effect, and indirect effect of the independent variable (copper) on the dependent variable (four lipids) through the mediator (hs-CRP). To ensure the robustness of the indirect effects, 95% confidence intervals were calculated using the bootstrap method. Multivariable models were adjusted by the covariables mentioned in “Relevant Covariates.” When performing regression analysis, to reduce the potential impact of extreme values on the analysis results, we used the 95th percentile of urinary copper concentration as the cutoff point to improve the robustness and accuracy of the analysis. Considering that systemic inflammation may also affect urinary copper excretion, we used structural equation modeling for sensitivity analysis to verify the robustness of the results.

Statistical significance was considered for a two-sided test with *p* < 0.05.

## Results

### Selection of Participants

**Fig. 1:**
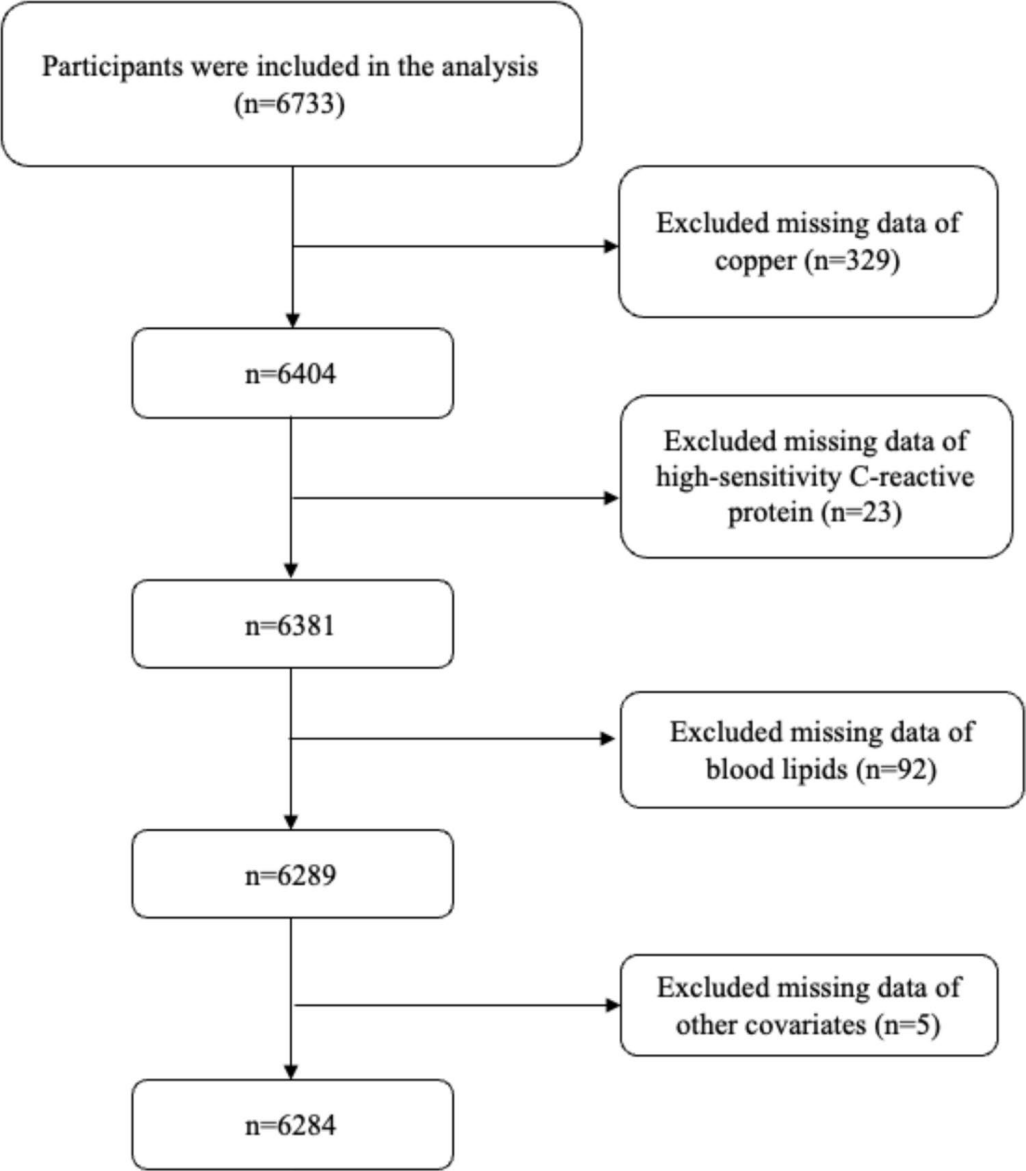
Selection of participants. CoLaus|PsyCoLaus study, Lausanne, Switzerland

We excluded participants with missing 1) copper data (*n* = 329); 2) hs-CRP data (*n* = 23); 3) blood lipids data (*n* = 92); and 4) covariates data (*n* = 5). Finally, 6284 participants were included in this analysis (Fig. 1). The characteristics of included and excluded participants are presented in Supplementary Table 1. Compared with the included participants, the excluded participants were less likely to be female, more likely to present with obesity and hypertension, had relatively higher TC and TG levels, and relatively lower HDL-C levels.

The characteristics of the included participants overall and by quartiles of urinary copper concentration are provided in Table 1. Among the participants selected for the study (mean age 52.6 years, 53.4% female), the mean plasma concentrations of TC, HDL-C, and LDL-C were 5.6 mmol/L, 1.6 mmol/L, and 3.3 mmol/L, respectively, and median plasma concentrations of TG and C-reactive protein were 1.1 mmol/L and 1.2 mg/L (Table 1). When copper concentration increased, TC, HDL-C, TG, and hs-CRP levels increased (all *p* < 0.05). In addition, increasing quartiles of urinary copper levels were associated with a higher frequency of older participants, females, those with lower educational attainment, living alone, obesity, hypertension, and diabetes (all *p* < 0.05). Conversely, the frequency of participants who consumed alcohol, smoked, and engaged in physical activity decreased across quartiles (all *p* < 0.05) (Table 1).

**Table 1:** Characteristics of the participants, according to quartiles of urinary copper levels

Variables	All participants	Quartiles of urinary copper concentration (μg/g creatinine)				*p*-value
		Quartile 1	Quartile 2	Quartile 3	Quartile 4	
Age (years)	52.6 ± 10.7	48.9 ± 9.6	51.3 ± 10.3	53.6 ± 10.6	56.6 ± 10.9	** < 0.001**
Women (%)	3354 (53.4)	540 (34.4)	787 (50.1)	957 (60.9)	1070 (68.1)	** < 0.001**
Educational level (%)						** < 0.001**
Low	3510 (55.9)	778 (49.6)	841 (53.6)	902 (57.6)	989 (63.1)	
Middle	1522 (24.3)	388 (24.8)	392 (25.0)	376 (24.0)	366 (23.4)	
High	1240 (19.8)	402 (25.6)	337 (21.4)	289 (18.4)	212 (13.5)	
Marital status (%)						** < 0.001**
Living alone	2080 (33.1)	436 (27.8)	491 (31.2)	538 (34.3)	615 (39.2)	
Living in couple	4198 (66.9)	1132 (72.2)	1080 (68.8)	1031 (65.7)	995 (60.8)	
Alcohol consumption (%)						** < 0.001**
None	1794 (28.5)	320 (20.4)	406 (25.8)	488 (31.1)	580 (36.9)	
1–13/week	3416 (54.4)	928 (59.1)	884 (56.3)	837 (53.3)	767 (48.8)	
14–27/week	848 (13.5)	257 (16.3)	226 (14.4)	190 (12.1)	175 (11.2)	
28 + /week	226 (3.6)	66 (4.2)	55 (3.5)	56 (3.5)	49 (3.1)	
Smoking categories (%)						**0.003**
Never	2562 (40.8)	594 (37.9)	623 (39.6)	654 (41.7)	691 (44.0)	
Former	2036 (32.4)	512 (32.6)	507 (32.3)	528 (33.6)	489 (31.2)	
Current	1681 (26.8)	463 (29.5)	441 (28.1)	387 (24.7)	390 (24.8)	
BMI categories (%)						** < 0.001**
Normal	3048 (48.5)	769 (48.9)	810 (51.6)	761 (48.4)	708 (45.1)	
Overweight	2282 (36.3)	611 (38.9)	568 (36.1)	560 (35.7)	543 (34.5)	
Obese	954 (15.2)	191 (12.2)	193 (12.3)	250 (15.9)	320 (20.4)	
Hypertension (%)	2311 (36.8)	424 (27.0)	496 (31.6)	596 (37.9)	795 (50.6)	** < 0.001**
Diabetes (%)	400 (6.4)	64 (4.1)	73 (4.7)	93 (5.9)	170 (10.8)	** < 0.001**
Physical activity (%)						** < 0.001**
Never	2209 (35.7)	517 (33.7)	517 (33.3)	562 (36.2)	613 (39.4)	
Once a week	609 (9.8)	181 (11.8)	174 (11.2)	140 (9.0)	114 (7.3)	
Twice a week	3284 (53.0)	819 (53.4)	835 (53.7)	834 (53.7)	796 (51.2)	
Does not know	95 (1.5)	17 (1.1)	28 (1.8)	18 (1.1)	32 (2.1)	
hs-CRP (mg/L)	1.2 [0.6–2.7]	1.0 [0.5–2.0]	1.1 [0.5–2.4]	1.4 [0.7–2.8]	1.8 [0.8–3.9]	** < 0.001**
Lipids (mmol/L)						
Total cholesterol	5.6 ± 1.0	5.5 ± 1.0	5.5 ± 1.0	5.6 ± 1.0	5.6 ± 1.1	**0.012**
HDL-C	1.6 ± 0.4	1.6 ± 0.4	1.6 ± 0.4	1.7 ± 0.4	1.7 ± 0.4	** < 0.001**
LDL-C	3.3 ± 0.9	3.3 ± 0.9	3.3 ± 0.9	3.3 ± 0.9	3.3 ± 1.0	0.997
Triglycerides	1.1 [0.8–1.6]	1.1 [0.8–1.6]	1.1 [0.8–1.6]	1.1 [0.8–1.5]	1.2 [0.9–1.7]	** < 0.001**

Results are expressed as number of participants (column percentage) for categorical variables and as average ± standard deviation or median [interquartile range] for continuous variables. Between-group comparisons were performed using chi-square for categorical variables and Student’s *t*-test or Kruskal–Wallis test for continuous variables

### Association Between Urinary Copper and Blood Lipids

As shown in Table 2, after adjusting for confounding factors, for each 1% increase in log-transformed urinary copper, there was a 0.04 mmol/L (95%CI − 0.07, − 0.003) decrease in HDL-C and an 8% (95% CI 4%, 12%) increase in TG. Similarly, when urinary copper levels were analyzed as quartiles, participants in the highest quartile had an 8% higher TG compared to those in the lowest quartile (95% CI 4%, 11%). However, TC, HDL-C, and LDL-C showed no significant differences across quartiles of urinary copper concentrations (95% CIs all crossed 0).

**Table 2:** Associations between urinary copper and blood lipids

Variables	Percent changes (95% CIs) by quartiles of urinary copper concentration				Each one-unit increase of log-transformed urinary copper	*p*-values
	Quartile 1	Quartile 2	Quartile 3	Quartile 4		
TC	0.00 (ref)	− 0.01 (− 0.07,0.06)	− 0.05 (− 0.12, 0.03)	− 0.02 (− 0.10, 0.06)	− 0.05 (− 0.14, 0.04)	0.279
HDL-C	0.00 (ref)	− 0.001 (− 0.03, 0.02)	− 0.03 (− 0.05, 0.001)	− 0.02 (− 0.05, 0.01)	− 0.04 (− 0.07, − 0.003)	**0.031**
LDL-C	0.00 (ref)	− 0.01 (− 0.08, 0.05)	− 0.03 (− 0.10, 0.03)	− 0.04 (− 0.11, 0.03)	− 0.07 (− 0.15, 0.02)	0.113
TG	0.00 (ref)	0.02 (− 0.01, 0.05)	0.01 (− 0.02, 0.04)	0.08 (0.04, 0.11)	0.08 (0.04, 0.12)	** < 0.001**

The multivariable associations were assessed using linear regression, results are expressed as percent changes (95% confidence interval), each one-unit increase (95% confidence interval), and the corresponding *p*-value. Regressions were adjusted for age, sex, BMI categories (normal, overweight, and obese), education (low/medium/high), marital status (alone and in a couple), smoking (never, former, and current), alcohol consumption (none, 1–13, 14–27, and 28 + per week), hypertension (yes and no), diabetes (yes and no), physical activity (never, once, or twice per week)

### Association Between Urinary Copper and Plasma hs-CRP

**Fig. 2:**
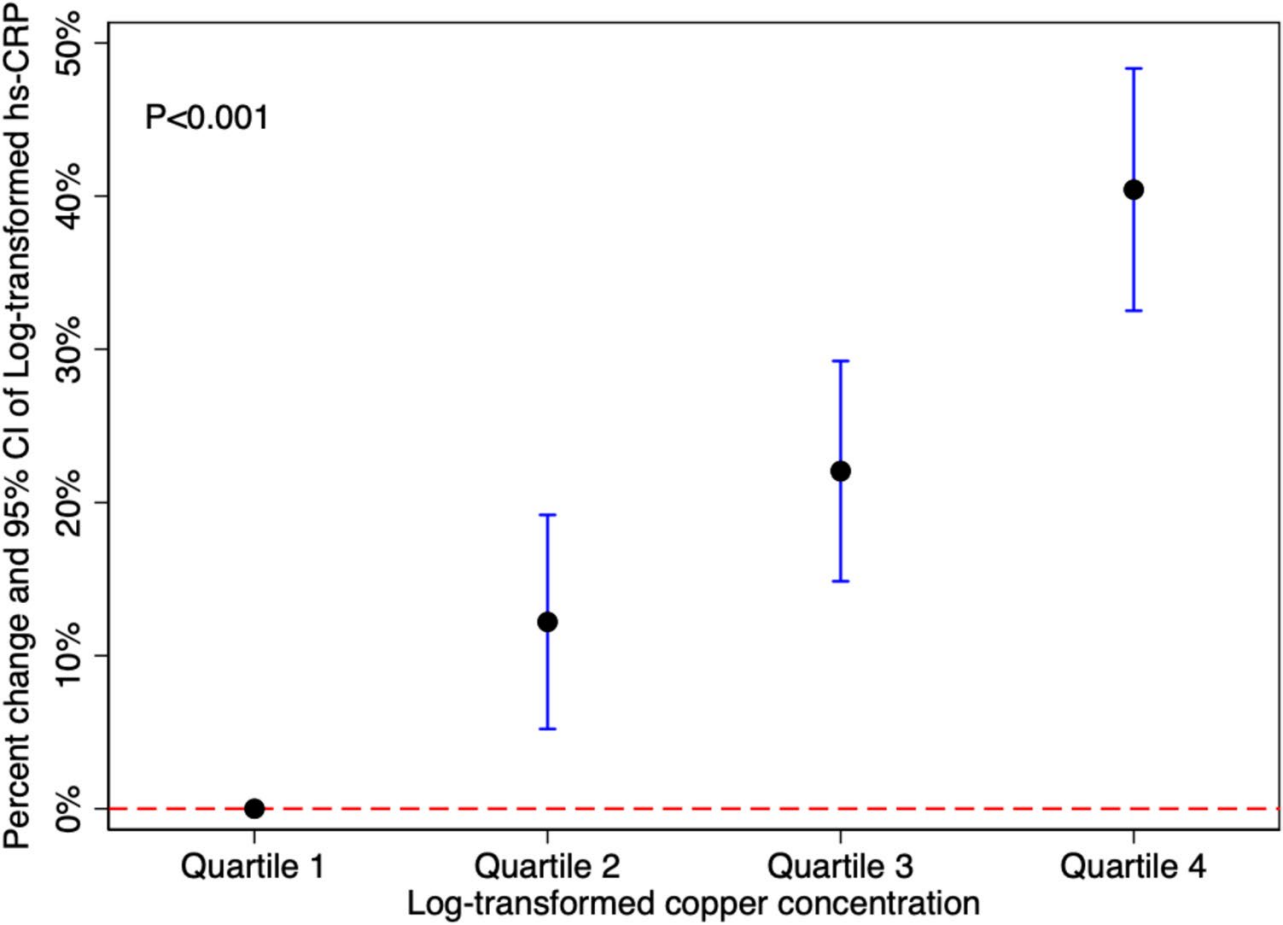
Urinary copper and plasma hs-CRP (urinary copper concentration adjusted for creatinine). CoLaus|PsyCoLaus study, Lausanne, Switzerland

As shown in Fig. 2, urinary copper was positively associated with plasma hs-CRP. For every 1% increase in urinary copper, the increase in plasma hs-CRP was 51% (95%CI 42%, 60%). Compared with the lowest quartile of urinary copper, the percentage change and 95% CI of plasma hs-CRP in the highest quartile was 40% (33%, 48%). Urinary copper without creatinine correction was positively associated with plasma hs-CRP in a dose–response relationship (Supplementary Fig. 1).

### Association Between Plasma hs-CRP and Blood Lipids

As shown in Table 3, after adjusting for confounding factors, the increase in plasma hs-CRP was significantly and positively associated with TG and significantly and negatively associated with HDL-C. For a 1% increase in plasma hs-CRP, TG increased by 5% and HDL-C decreased by 4%. No association between plasma hs-CRP and TC or LDL-C was observed. This trend was also confirmed in the quartile analysis. Compared to the lowest quartile of hs-CRP, the highest quartile was significantly associated with higher TG and lower HDL-C, while no significant associations were observed for TC or LDL-C.

**Table 3:** Associations between plasma hs-CRP and blood lipids

Variables	Percent changes (95% CIs) by quartiles of plasma hs-CRP concentration (mg/L)				Each one-unit increase of log-transformed plasma hs-CRP concentration (mg/L)	*p*-values
	Quartile 1	Quartile 2	Quartile 3	Quartile 4		
TC	0.00 (ref)	0.11 (0.04, 0.18)	0.09 (0.02, 0.16)	− 0.001 (− 0.08, 0.07)	− 0.01 (− 0.03, 0.02)	0.597
HDL-C	0.00 (ref)	− 0.03 (− 0.05, − 0.003)	− 0.08 (− 0.11, − 0.06)	− 0.11 (− 0.14, − 0.08)	− 0.04 (− 0.05, − 0.03)	** < 0.001**
LDL-C	0.00 (ref)	0.10 (0.04, 0.16)	0.10 (0.04, 0.16)	0.03 (− 0.04, 0.09)	0.01 (− 0.01, 0.03)	0.487
TG	0.00 (ref)	0.08 (0.05, 0.11)	0.13 (0.10, 0.16)	0.15 (0.12, 0.19)	0.05 (0.04, 0.06)	** < 0.001**

The multivariable associations were assessed using linear regression. Results are expressed as percent changes (95% confidence interval), each one-unit increase (95% confidence interval), and the corresponding *p*-value. Regressions were adjusted for age, sex, BMI categories (normal, overweight, and obese), education (low/medium/high), marital status (alone and in a couple), smoking (never, former, and current), alcohol consumption (none, 1–13, 14–27, and 28 + per week), hypertension (yes and no), diabetes (yes and no), physical activity (never, once, or twice per week)

### Mediating Effect of hs-CRP on the Association Between Urinary Copper and Blood Lipids

As shown in Table 4, hs-CRP demonstrated a potential mediating effect in the association between urinary copper and HDL-C and TG, though the statistical significance and confidence levels varied. For the association between urinary copper and HDL-C, the proportion of mediation was 56.9%, with a *p*-value < 0.001 for the indirect effect. However, the Bootstrap confidence interval ranged from − 13.48 to 14.62, including 0, indicating uncertainty in the statistical significance of the mediation effect despite its apparent significance. In contrast, for the association between urinary copper and TG, the proportion of mediation was 31.4%, with a robust Bootstrap confidence interval of 7.1 to 55.6% and a *p*-value < 0.001, suggesting a significant and reliable mediation effect. On the other hand, no significant mediating effect of hs-CRP was observed in the associations between urinary copper and TC or LDL-C, with indirect effect *p*-values of 0.495 and 0.454, respectively, and confidence intervals including 0. Overall, hs-CRP partially mediated the associations between urinary copper and HDL-C and TG, with a stronger and more statistically reliable effect for TG. However, the mediating effect on HDL-C should be interpreted with caution.

**Table 4:** Mediation effects of hs-CRP on the association between copper and blood lipids

Variables	Total effects, Est	Direct effects, Est	Indirect effects, Est	Proportion mediation and 95% CI	*p-*value
TC	− 0.050	− 0.045	− 0.005	-	0.495
HDL-C	− 0.037	− 0.016	− 0.021	0.569 (− 13.48,14.62)	** < 0.001**
LDL-C	− 0.065	− 0.070	0.004	-	0.454
TG	0.077	0.053	0.024	0.314 (0.071,0.556)	** < 0.001**

Results are expressed as mediation ratios (95% CI) and corresponding *p*-values. Models were adjusted for age, sex, BMI categories (normal, overweight, and obese), education (low/medium/high), marital status (alone and in a couple), smoking (never, former, and current), alcohol consumption (none, 1–13, 14–27, and 28 + per week), hypertension (yes and no), diabetes (yes and no), physical activity (never, once, or twice per week)

## Discussion

This study revealed the potential mediating role of hs-CRP in the association between urinary copper and lipid levels. Specifically, hs-CRP partially mediated the association between urinary copper and HDL-C and TG. The mediation effect for TG demonstrated strong statistical significance and robustness, whereas the mediation effect for HDL-C, although significant, had a confidence interval that included zero, indicating statistical uncertainty. Additionally, no significant mediating role of hs-CRP was observed in the associations between urinary copper and TC or LDL-C. These findings suggest that hs-CRP may play a role in the pathophysiological processes underlying lipid metabolism disorders, particularly in the regulation of HDL-C and TG. However, further research is needed to validate these findings.

Epidemiological studies have shown that exposure to high copper concentrations is associated with increased blood lipids and cardiovascular disease risk [8–10, 20]. However, the specific mechanisms are still unclear. Previous studies have reported a positive association between copper and hs-CRP [14–16]. Excessive copper can cause oxidative stress, thereby triggering inflammation [21]. In addition, multiple studies have found that excessive exposure to copper can activate the pyrin domain-containing protein 3 (NLRP3) inflammasome [22–24]. This activation mechanism is partly because copper ions significantly trigger cellular oxidative stress, thereby mediating the activation of the NLRP3 inflammasome [22]. The study also found that the activation of the NLRP3 inflammasome can be effectively blocked by removing copper from the active site of superoxide dismutase 1 (SOD1) [23]; at the same time, significant activation of the NLRP3 inflammasome was also observed in experiments in which primary microglia were treated with CuCl_2_ [24]. On the other hand, hs-CRP is associated with dyslipidemia [25], positively correlated with TG, and negatively correlated with HDL-C [26].

While excessive copper exposure has been linked to increased cardiovascular risk, copper deficiency may also contribute to lipid dysregulation. Epidemiological studies have shown that plasma copper levels are reduced in patients with coronary artery disease and hyperlipidemia [27]. Animal studies further support this association, as rats fed a copper-deficient diet exhibited elevated plasma cholesterol, triglycerides, and uric acid levels [28]. The mechanisms linking copper deficiency to dyslipidemia remain unclear, but proposed pathways involve its role as a cofactor for enzymes like superoxide dismutase and ceruloplasmin, which regulate oxidative stress and cholesterol transport [29]. Copper deficiency may impair these processes, leading to oxidative stress, inflammation, and lipid abnormalities [30]. These findings underscore the dual role of copper in lipid metabolism, where both deficiency and excess can disrupt lipid homeostasis.

Our study measured urinary copper, which reflects the state of copper exposure to a certain extent. Compared with blood copper, urinary copper is not easily affected by multiple factors such as diet or infection [31]. We found that urinary copper was significantly and positively associated with TG and negatively associated with HDL-C, which is in agreement with the literature [10]. For instance, the results of a study conducted in China by Ma et al. showed that increased urinary copper was significantly and positively associated with TG and that hs-CRP partially mediated the relationship between urinary copper and TG [10]. Interestingly, in our study, besides TG, we also observed that hs-CRP may partially mediate the negative association between urinary copper and HDL-C. However, although the *p*-value for the indirect effect was statistically significant, the Bootstrap confidence interval included zero, indicating uncertainty in the statistical significance of this mediating effect. Therefore, the specific role of hs-CRP in the association between urinary copper and HDL-C requires further investigation and validation.

### Strengths and Limitations

To our knowledge, this is the first study assessing the associations between urinary copper and lipid levels in a European population. Our results thus generalize those of Ma et al.’s study to ethnic groups other than Chinese. Further, our sample size is larger (6284 vs. 3301), which improves the statistical power and makes the estimates more accurate.

This study also has some limitations. First, it is possible that the association between urinary copper and HDL-C is influenced by diet. Still, as urinary copper is not easily affected by multiple factors such as diet, we believe that this hypothesis is unlikely. Second, the absence of blood copper measurements limits our ability to fully assess systemic copper metabolism. Future studies incorporating both blood and urinary copper measurements may provide deeper insights into the relationship between copper, lipid metabolism, and inflammation.

## Conclusion

High urinary copper levels are unfavorably associated with blood lipids, especially TG and HDL-C. hs-CRP partially mediates the link between urinary copper and related lipid levels, but the specific mediating mechanism is still unclear.

## Supplementary Information

Below is the link to the electronic supplementary material.

Supplementary file1 (DOCX 68 KB)

## Data Availability

The data of CoLaus|PsyCoLaus study used in this article cannot be fully shared as they contain potentially sensitive personal information on participants. According to the Ethics Committee for Research of the Canton of Vaud, sharing these data would be a violation of the Swiss legislation with respect to privacy protection. However, coded individual-level data that do not allow researchers to identify participants are available upon request to researchers who meet the criteria for data sharing of the CoLaus|PsyCoLaus Datacenter (CHUV, Lausanne, Switzerland). Any researcher affiliated to a public or private research institution who complies with the CoLaus|PsyCoLaus standards can submit a research application to research.colaus@chuv.ch or research.psycolaus@chuv.ch. Proposals requiring baseline data only, will be evaluated by the baseline (local) Scientific Committee (SC) of the CoLaus and PsyCoLaus studies. Proposals requiring follow-up data will be evaluated by the follow-up (multicentric) SC of the CoLaus|PsyCoLaus cohort study. Detailed instructions for gaining access to the CoLaus|PsyCoLaus data used in this study are available at www.colaus-psycolaus.ch/professionals/how-to-collaborate/.

## Declarations

### Disclaimer

The funders had no role in the design of the study; in the collection, analyses, or interpretation of data; in the writing of the manuscript; or in the decision to publish the results.
