# Supplementary material for: Urinary Copper Is Associated with Dyslipidemia, and This Association Is Mediated by Inflammation
Source: Biol Trace Elem Res. 2025 Apr 2;203(11):5505–14. doi: 10.1007/s12011-025-04581-6 (PMC12602554; doi:10.1007/s12011-025-04581-6)
Supplement: Supplementary file 1 — Supplementary file1 (DOCX 68 KB) [file 12011_2025_4581_MOESM1_ESM.docx]

**Supplementary Material**

**Article Title:** Urinary copper is associated with dyslipidemia, and this association is mediated by inflammation

**Journal Name:** Biological Trace Element Research

**Authors:** Sisi Xie, Zoltan Kutalik Aurélien Thomas, Maiwenn Perrais, Julien Vaucher, and Pedro Marques-Vidal

**Corresponding Author:** Sisi.Xie@unil.ch

**Supplementary Figure 1:** Urinary copper and plasma hs-CRP (Urinary copper concentration not adjusted for creatinine).

**
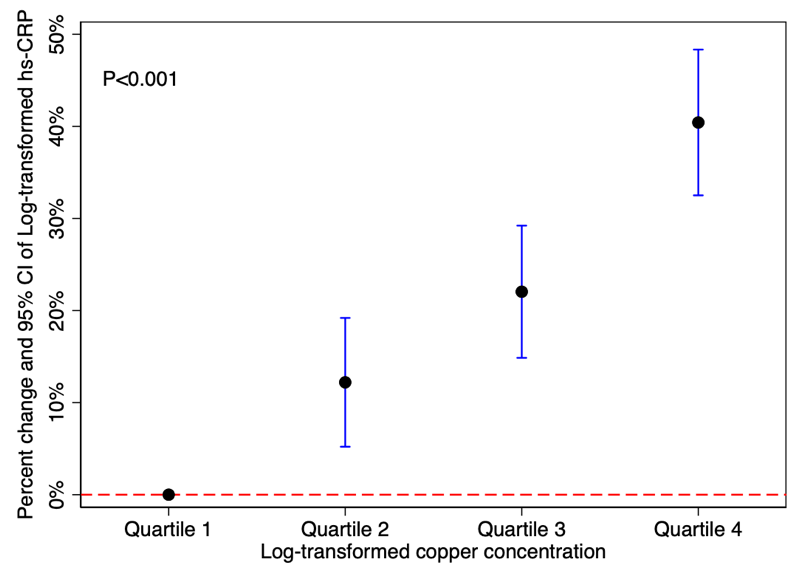
**

**Supplementary Table 1:** Characteristics of included and excluded participants.

|  | **Included**  (n=6284) | **Excluded**  (n=449) | **P-value** |
| --- | --- | --- | --- |
| Age (years) | 52.6 ± 10.7 | 53.1 ± 10.9 | 0.328 |
| Women (%) | 3354 (53.4) | 190 (42.3) | **<0.001** |
| Educational level (%) |  |  | 0.424 |
| Low | 3510 (55.9) | 264 (59.1) |  |
| Middle | 1522 (24.3) | 103 (23.0) |  |
| High | 1240 (19.8) | 80 (17.9) |  |
| Marital status (%) |  |  | 0.161 |
| Living alone | 2080 (33.1) | 134 (29.9) |  |
| Living in couple | 4198 (66.9) | 314 (70.1) |  |
| Alcohol consumption (%) |  |  | 0.100 |
| None | 1794 (28.5) | 121 (26.9) |  |
| 1-13/week | 3416 (54.4) | 237 (52.8) |  |
| 14-27/week | 848 (13.5) | 65 (14.5) |  |
| 28+/week | 226 (3.6) | 26 (5.8) |  |
| Smoking categories (%) |  |  | 0.405 |
| Never | 2562 (40.8) | 170 (38.0) |  |
| Former | 2036 (32.4) | 147 (32.8) |  |
| Current | 1681 (26.8) | 131 (29.2) |  |
| BMI categories (%) |  |  | **0.043** |
| Normal | 3048 (48.5) | 189 (42.4) |  |
| Overweight | 2282 (36.3) | 180 (40.4) |  |
| Obese | 954 (15.2) | 77 (17.2) |  |
| Hypertension (%) | 2311 (36.8) | 189 (42.8) | **0.012** |
| Diabetes (%) | 400 (6.4) | 36 (8.3) | 0.111 |
| Physical activity (%) |  |  | 0.602 |
| Never | 2209 (35.7) | 168 (38.0) |  |
| Once a week | 609 (9.8) | 42 (9.5) |  |
| Twice a week | 3284 (53.0) | 223 (50.5) |  |
| Does not know | 95 (1.5) | 9 (2.0) |  |
| hs-CRP, mg/L | 1.2 [0.6-2.7] | 1.5 [0.7-2.7] | 0.113 |
| Lipids, mmol/L |  |  |  |
| Total cholesterol | 5.6 ± 1.0 | 5.9 ± 1.2 | **<0.001** |
| HDL-C | 1.6 ± 0.4 | 1.5 ± 0.5 | **<0.001** |
| LDL-C | 3.3 ± 0.9 | 3.3 ± 1.0 | 0.955 |
| Triglycerides | 1.1 [0.8-1.6] | 1.3 [0.9-3.0] | **0.001** |

Results are expressed as number of participants (column percentage) for categorical variables and as average ± standard deviation or median [interquartile range] for continuous variables. Between-group comparisons were performed using chi-square for categorical variables and student’s t-test or Kruskal-Wallis test for continuous variables.
